# A feasibility study to inform the design of a randomized controlled trial to identify the most clinically and cost effective Anticoagulation Length with low molecular weight heparin In the treatment of Cancer Associated Thrombosis (ALICAT): study protocol for a mixed-methods study

**DOI:** 10.1186/1745-6215-15-122

**Published:** 2014-04-12

**Authors:** Joanna D Smith, Jessica Baillie, Trevor Baglin, Gareth O Griffiths, Angela Casbard, David Cohen, David A Fitzmaurice, Kerenza Hood, Peter Rose, Alexander T Cohen, Miriam Johnson, Anthony Maraveyas, John Bell, Harold Toone, Annmarie Nelson, Simon I Noble

**Affiliations:** 1 Wales Cancer Trials Unit, Cardiff University School of Medicine, Heath Park, Cardiff, Wales CF14 4YS, UK; 2 Marie Curie Palliative Care Research Centre, Cardiff University School of Medicine, Heath Park, Cardiff, Wales CF14 4YS, UK; 3 Department of Hematology, Addenbrooke's Hospital, Cambridge University Hospitals NHS, Foundation Trust, Hills Road, Cambridge CB2 0QQ, UK; 4 Faculty of Health, Sport and Science, University of South Wales, Pontypridd CF37 1DL, UK; 5 Primary Care Clinical Sciences, School of Health and Population Sciences, College of Medical and Dental Sciences, University of Birmingham, Edgbaston, Birmingham B15 2TT, UK; 6 South East Wales Trials Unit, Cardiff University School of Medicine, Heath Park, Cardiff, Wales CF14 4YS, UK; 7 Department of Hematology, Warwick Hospital, Lakin Road, Warwick CV34 5BW, UK; 8 Department of Surgery and Vascular Medicine, King’s College Hospital, Denmark Hill, London SE5 9RS, UK; 9 Hull York Medical School, The University of Hull, Castle Road, Hull HU16 5JQ, UK

**Keywords:** Venous thromboembolism, Pulmonary embolus, Deep vein thrombosis, Cancer associated thrombosis, Low molecular weight heparin, Randomized controlled trial, Mixed methods, Palliative care, Qualitative research, Framework analysis

## Abstract

**Background:**

Venous thromboembolism is common in patients with cancer and requires anticoagulation with low molecular weight heparin. Current data informs anticoagulation as far as six months, yet guidelines recommend anticoagulation beyond six months in patients who have locally advanced or metastatic cancer. This recommendation, based on expert consensus, has not been evaluated in a clinical study. ALICAT (Anticoagulation Length in Cancer Associated Thrombosis) is a feasibility study to identify the most clinically and cost effective length of anticoagulation with low molecular weight heparin in the treatment of cancer associated thrombosis.

**Methods/Design:**

ALICAT is a randomized multi-centre phase two mixed-methods study with three components: a randomized controlled trial, embedded qualitative study and a survey investigating pathways of care. The randomized controlled trial will compare ongoing low molecular weight heparin treatment for cancer-associated thrombosis versus cessation of low molecular weight heparin at six months treatment (current licensed practice) in patients with locally advanced or metastatic cancer. The embedded qualitative study will include focus groups with clinicians to investigate attitudes to recruiting to the study, identify the challenges of progressing to a full randomized controlled trial, and also semi-structured interviews with patients and relatives/carers to explore their attitudes towards participating in the study and potential barriers and concerns to participation. Finally, a UK wide survey exercise will be undertaken to develop a classification and enumeration system for the cancer associated thrombosis models and pathways of care.

**Discussion:**

There is a lack of evidence determining the length of anticoagulation for patients with cancer associated thrombosis and subsequently treatment length varies. The ALICAT study will consider the feasibility of recruiting patients to a phase three trial.

**Trial registration:**

Current Controlled Trials ISRCTN37913976.

## Background

Venous thromboembolism (VTE), comprising of deep vein thrombosis (DVT) and pulmonary embolus (PE), is a common phenomenon which occurs in 1 in 1,000 patients and annually affects 6.5 million people worldwide [1,2]. Within the UK, the cost of managing VTE has been estimated at £640 million per year, and the prevention of VTE has become a health service priority [3]. Individuals with cancer are at particular risk of VTE due to the local release of procoagulants, such as tissue factor, and the prothrombotic nature of oncological treatments including surgery, chemotherapy and radiotherapy [4]. Up to 18% of patients with cancer will develop VTE, requiring anticoagulation [4]. The standard treatment of VTE is well established, consisting of five days anticoagulation with low molecular weight heparin (LMWH), followed by three to six months warfarin [4,5]. However, the management of cancer associated thrombosis (CAT) presents several challenges due to a higher rate of both re-thrombosis and bleeding amongst patients with cancer when compared to those with a non-malignant disease [6].

The impact of VTE on patients with cancer is substantial; conferring a worse prognosis when compared with similar stage cancer patients without VTE [7,8]. Furthermore, anticoagulation with warfarin is complicated by drug-drug interactions, variable drug absorption, and changing nutritional status [9]. This inevitably has a practical impact on the delivery of anti-cancer therapies. Maintaining stable coagulation with warfarin is difficult and requires more frequent monitoring with blood tests, which adversely affects patients’ quality of life [10]. Three randomized controlled trials (RCTs) have compared LMWH with warfarin in the treatment of CAT, demonstrating superior efficacy with LMWH without statistical difference in bleeding complications [11-13]. Consequently, national and international clinical guidelines recommend the gold standard treatment of CAT to be six months treatment with weight adjusted LMWH [6,14-16].

The management of CAT beyond six months is less clear; in patients with locally advanced or metastatic cancer there is an ongoing prothrombotic state which may warrant anticoagulation beyond the recommended period. In support of indefinite anticoagulation are data suggesting the prothrombotic risk increases with disease progression, due to a greater tumor burden, reduced performance status and increased use of palliative chemotherapy [17-19]. However, with cancer progression also comes an increased bleeding risk during anticoagulation, and the decision to anticoagulate indefinitely will need to balance the benefits of preventing recurrent VTE against the risks of major bleeding [20,21]. Furthermore, it is important to consider the impact of long term LMWH use on patients’ quality of life.

Currently, clinical guidelines recommend clinicians consider continuing anticoagulation indefinitely in patients with locally advanced or metastatic cancer [15,16,22]. Such recommendations are based largely on consensus opinion and, to date, have been supported by limited clinical evidence beyond case series and observational studies [23,24]. In the recently reported DALTECAN (Dalteparin sodium for the long-term management of venous thromboembolism in cancer patients) study, 334 patients with VTE and locally advanced or metastatic cancer were treated with dalteparin, of whom 185 (55.4%) completed six months of therapy and 109 (33%) completed twelve months [24]. The authors reported a 10.2% overall frequency of major bleeding and an overall incidence of new or recurrent VTE of 11.1%. In patients treated during the extended anticoagulation period, new or recurrent VTE was reported in 4.1% of patients and major bleeding at a rate of 4.2%. It is notable that both bleeding and recurrent thrombosis rates were higher in the first six months of anticoagulation (1.3% and 1.4% per patient-month respectively) compared with months seven to twelve (0.7% and 0.7% per patient-month respectively) [24]. In the absence of a control arm in which patients received placebo/ceased anticoagulation at six months, the study offers no further guidance as to whether anticoagulation beyond six months provides any net benefit. However, it offers valuable data, which can be used to inform future clinical studies. Firstly, with 96% trial adherence, it suggests that the use of LMWH beyond six months is feasible within the trial setting. Secondly, it suggests that safety concerns regarding bleeding in this patient group may be less of an issue than previously believed. Furthermore, with the rate of VTE recurrence being lower during the extended anticoagulation period than the initial six months, it would seem reasonable to question what degree of added clinical benefit is gained through extended treatment, rather than ceasing anticoagulation at six months [24]. Finally, the trial did not formally evaluate quality of life aspects of LMWH administration over an extended time period.

Several clinical studies are currently attempting to clarify CAT management beyond six months anticoagulation. In the LONGHEVA (Long-term Treatment for Cancer Patients With Deep Venous Thrombosis or Pulmonary Embolism) [25] study patients who have completed six to twelve months of treatment for VTE and still have an indication for further anticoagulation (metastatic cancer and/or ongoing cancer treatment) will be randomized to receive a further six months of vitamin K antagonist (VKA) or LMWH. The primary efficacy outcome is recurrence of confirmed symptomatic DVT with secondary outcomes including safety. Driven by the belief that patients with metastatic cancer or receiving cancer treatment require extended anticoagulation, the investigators seek to establish the most efficacious anticoagulant between VKAs and LMWHs [25]. Whilst the driving hypothesis feels intuitively sensible, this belief is yet to be quantified within the trial setting. Without robust data identifying the VTE risk of *not* continuing anticoagulation it becomes conceptually challenging to justify a therapeutic intervention that is yet to be proven beneficial.

SELECT-D (Anticoagulation therapy in SELECTeD cancer patients at risk of recurrence of venous thromboembolism.) is a pilot study comparing dalteparin versus rivoraxaban in the treatment of CAT with a second placebo-controlled randomization comparing the duration of anticoagulation therapy (six months versus twelve months treatment) in residual vein thrombosis [RVT] positive patients [26]. It is wholly appropriate that this study is piloted before proceeding to a full RCT since several key factors within the study design will need clarification in advance. Firstly, the investigators have chosen to compare dalteparin with rivaroxaban, a drug that is yet to demonstrate non-inferiority in the CAT setting. Secondly, the utility of RVT as a predictor of VTE recurrence specifically in the cancer-associated thrombosis setting is yet to be established [27,28].

It is arguable that when considering anticoagulation beyond six months, one should quantify the clinical need for prolonged anticoagulation before evaluating the most appropriate drug. Scientifically, such an approach has merit since it informs future studies from a basis of fact and not supposition. However, clinical practice has developed in such a way that many clinicians will continue anticoagulation regardless of the evidence deficit. For this reason, a study to identify whether patients with CAT and ongoing cancer should cease or continue anticoagulation at six months may be difficult to recruit to, since such practice feels counter intuitive to clinicians. Whether such a study would be feasible regardless of scientific merit is unclear. The authors therefore propose such a study to identify the feasibility of conducting a RCT comparing six months LMWH with indefinite anticoagulation in CAT patients with ongoing malignancy.

### Research aim and objectives

The ALICAT (Anticoagulation Length in Cancer Associated Thrombosis) study was developed in response to a National Institute for Health Research (NIHR) Health Technology Assessment (HTA) commissioned call considering duration of treatment of venous thromboembolism in malignant disease (reference: 10/145), to address a specific gap in the evidence base for the management of CAT in patients with ongoing malignant disease.

The aim of this study is to examine the feasibility of conducting a phase three randomized controlled trial determining the length of anticoagulation for patients with cancer associated thrombosis.

The objectives are to: (1) Identify the practicalities of conducting a full RCT with regard to recruitment, retention and outcome measurement, specifically: the number of eligible patients that can be recruited within a one year timeframe, the dropout rate, the practical utility of measuring primary outcome measures, reporting processes, and assessment tools within the context of a full RCT. (2) Explore the logistical and attitudinal barriers to progressing to a full RCT, in particular: how and where to identify patients for recruitment, the attitudes of clinicians towards entering patients on to the ALICAT trial and prescribing anticoagulation for patients with CAT, patients’ attitudes to taking part in the trial, including individuals who consented, those who refused to participate and people who withdrew post-randomization.

## Methods/Design

### Study design

**Figure 1:**
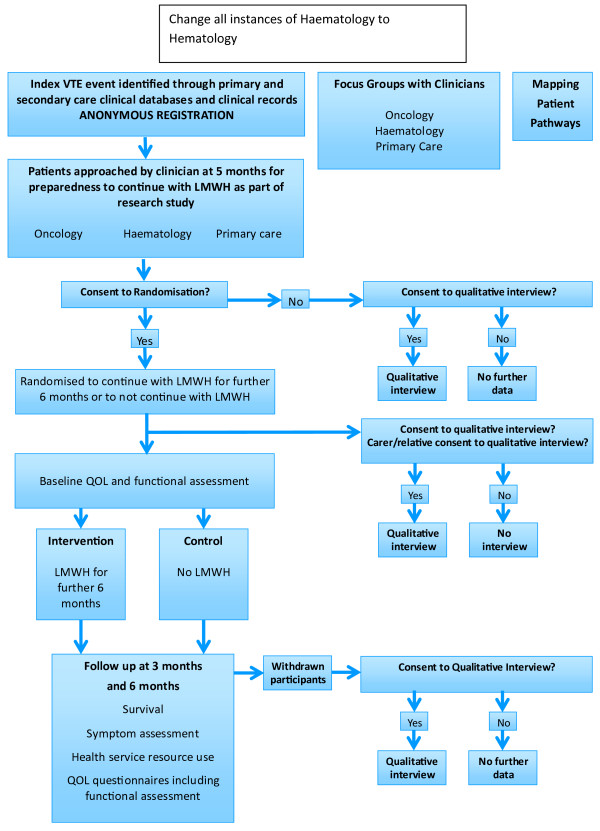
Trial schema.

The ALICAT trial schema is summarized in Figure 1. ALICAT is a mixed-methods study involving three components: a RCT, an embedded qualitative study that will include focus groups with clinicians and interviews with patients and their relatives, and a UK wide survey exercise to map patient pathways. Each component of the study is explained below.

### Randomized controlled trial

This component of the study comprises an open-label two arm RCT comparing ongoing LMWH treatment for CAT versus cessation of LMWH at six months treatment (current licensed practice) in patients with locally advanced or metastatic cancer.

#### Participant recruitment

In order to assess the feasibility of recruiting sufficient numbers of patients to inform a full phase three RCT, the ALICAT study will evaluate recruitment of patients from three different care settings within the UK: oncology outpatients, hematology outpatients, and primary care. Index VTE events shall be identified through primary and secondary care clinical databases and clinical records. In the oncology and hematology outpatient settings, potential participants will already be attending outpatient clinics for management of their cancer or CAT, and potential participants will be screened by a researcher and notified to the site principal investigator. Within the primary care setting, electronic patient records will be used to identify patients receiving treatment doses of LMWH. These records will be screened for patient eligibility of registration, by a researcher based in a primary care research institution. Patients who meet the eligibility criteria will be invited to the participating practice in order to consider participating in the study. The search will be repeated monthly in order to identify incident cases.

Potential patients will be identified when they are approaching completion of their first five months treatment with LMWH for CAT. Suitable patients will be anonymously registered. After written informed consent has been obtained, a screening baseline assessment will be conducted to ensure all the inclusion and exclusion criteria are met (see Table 1). Patients who meet the eligibility criteria will be randomized by the coordinating trials unit to either the treatment arm (continue LMWH treatment for a further six months) or the control arm (discontinue LMWH), using block randomization with varying block sizes and a 1:1 allocation ratio. Randomized patients will be asked to complete three baseline quality of life questionnaires (EORTC QLQ-C30, E5D-5 L and ESAS-r), and to keep a diary card during the six month follow up period, irrespective of the trial arm assigned.

**Table 1:** Inclusion/exclusion criteria and initial assessment for the ALICAT trial

**Inclusion criteria**	1. Receiving LMWH for treatment of CAT for approximately five months
	2. Locally advanced or metastatic cancer
	3. Able to self-administer LMWH, or have LMWH administered by a carer (routine administration by a district nurse is not permissible)
	4. Able to give informed consent
	5. Age ≥16 years.
**Exclusion criteria**	1. Receiving drug other than LMWH for CAT
	2. Contraindication to continuing anticoagulation:
	a. Known allergies to LMWHs, heparin, sulfites or benzyl alcohol,
	b. Active major bleeding,
	c. History of heparin-induced thrombocytopenia,
	d. Known poor compliance with LMWH,
	3. Confirmed recurrent VTE whilst receiving anticoagulation
	4. Fitted with a prosthetic heart valve.
**Initial assessment**	1. Full blood count, urea, electrolytes, liver function, bone profile
	2. Physical examination
	3. Primary tumor and tumor treatment history
	4. Details of LMWH treatment off-trial
	5. Baseline concomitant medications
	6. Baseline comorbidities (including index VTE and bleeding event history)
	7. Use of NHS resources three months prior to commencing trial treatment.

CAT, Cancer associated thrombosis; LMWH, Low molecular weight heparin; NHS, National Health Service; VTE, Venous thromboembolism.

Information on all potentially eligible patients will be recorded, including whether or not patients were actually eligible for the trial, if they were approached about the trial, if they agreed to randomization, or were approached about taking part in a qualitative interview. Where possible, information on why the patient was not approached, randomized or approached for the qualitative interview will also be recorded.

#### Trial treatment arm

Participants randomized to the treatment arm will have already received LMWH (dalteparin (Fragmin^®^), enoxaparin (Clexane^®^), or tinzaparin (Innohep^®^)) at the treatment dose for six months. As this is a pragmatic feasibility study, participating clinicians’ treatment practices will not be altered and participants will continue with the same LMWH. LMWH will be given as a daily subcutaneous dose at the same dose and time as previously administered over the previous six months. Dosing levels will be guided by the Summary of Product Characteristics (SPC) as described in Table 2.

**Table 2:** Recommended LMWH dose levels

**LMWH**	**Month one**	**Month two to six**	**Entry to ALICAT**
Dalteparin (Fragmin^®^)	200 International Units/kg total body weight, once daily	150 International Units/kg total body weight, once daily	Dose as detailed at month six
Tinzaparin (Innohep^®^)	175 International Units/kg total body weight, once daily	175 International Units/kg total body weight, once daily	Dose as detailed at month six
Enoxaparin (Clexane^®^)	150 International Units/kg total body weight, once daily	150 International Units/kg total body weight, once daily	Dose as detailed at month six

ALICAT, Anticoagulation Length with low molecular weight heparin In the treatment of Cancer Associated Thrombosis; LMWH, Low molecular weight heparin; kg, kilogram.

Switching from one ALICAT Investigational Medicinal Product (IMP) to another during the trial treatment period is allowed if deemed necessary by the treating clinician. Within current practice, the dose of LMWH may be altered at the discretion of the supervising clinician as described in Table 3. Any alteration of LMWH administration or dose and any episodes of bleeding or recurrent VTE will be documented.

**Table 3:** Permissible LMWH dose alterations

**Reason for increasing dose of LMWH:**	Recurrence of symptomatic VTE despite administration of weight adjusted dose as per SPC. Some clinicians may use measurements of anti-Xa levels to guide LMWH dosing in this situation.
**Reason for decreasing dose:**	Sometimes patients may experience minor bleeding which will resolve on decreasing the dose of LMWH. This is a clinical decision of the supervising clinician that will be made based on balancing the perceived risks of recurrent VTE should LMWH be stopped or major bleeding should LMWH be continued.
**Dose for obese patients:**	Follow the SPC or local clinical practice.
**Dose for renal impairment:**	Follow the SPC. Patients with renal impairment should have dose adjustment depending on creatinine clearance/anti factor Xa levels according to local policy.

LMWH, Low molecular weight heparin; SPC, summary of product characteristics; VTE, venous thromboembolism.

#### LMWH supply

As ALICAT is a pragmatic trial, it is not the intention to change the way the LMWH is normally prescribed. A clinical trial labelling exemption has been obtained from the Medicines and Healthcare products Regulatory Authority (MHRA) and the trial will be fully exempt from the UK statutory instrument The Medicines for Human Use (Clinical Trials) Regulations 2004 No. 1031 Part 7 Regulation 46 with respect to IMP labelling and dispensing. The LMWH will be prescribed by the participant’s general practitioner (GP), oncologist or hematologist according to pre-existing local arrangements. The drug will be dispensed from commercial stock through local hospital pharmacies and community pharmacies. It is anticipated that excess treatment costs will only be incurred for participants allocated to the treatment arm at participating sites where currently it is not common practice to extend LMWH treatment for CAT beyond six months. Excess treatment costs will not be incurred for participants allocated to the control arm of the trial, irrespective of the local policy for extended LMWH treatment.

#### RCT sample size calculation

We anticipate there being at least 200 eligible patients per year in total from all three recruitment settings. We assume that not all patients who have been injecting LMWH for five months will agree to continue for a further six months. We are not sure what proportion of patients will be willing to be randomized, but we consider that at least 30% will need to agree in order to make a phase three RCT worthwhile. Therefore, the ALICAT study will aim to assess the feasibility of randomization by determining if at least 30% of potential patients will agree to randomization. We will then calculate the precision of this proportion with a 95% confidence interval. We will also estimate the proportion of patients who experience recurrent VTEs during follow up, which will inform the design of the phase three RCT.

If less than 15% of eligible patients agree to take part in the trial, a phase two RCT may not be suitable as we may not be able to recruit suitable numbers, given that a larger phase three RCT could have more stringent eligibility criteria as a result of this feasibility study. If at least 30% agree to take part, then we would consider that randomizing patients to a larger phase three trial would be feasible. Using a Fleming’s single stage design, setting p1 to 0.15 and p2 to 0.3, and with 5% significance and 90% power, then we will need to approach 62 eligible participants with details of the trial. This design requires that at least 15 out of 62 participants consent to the trial. We therefore propose a two-stage sample size. If at least 15 out of the first 62 participants recruited to the trial accept randomization, then we will continue recruitment into stage two. If fewer than 15 patients agree, we will determine at this stage that randomization within this population is not feasible.

If we expect that 200 will be the maximum potential number of eligible patients, then we can expect to be able to produce a 95% confidence interval for the percentage willing to be randomized of width 13.7% or less. For example, if the percentage is 50%, then we could expect the 95% confidence interval to be 43.15 to 56.85%. If the percentage is 30% we can expect a slightly smaller confidence interval of 23.7 to 36.3%. If only 150 patients are deemed eligible, then we can expect to calculate a 95% confidence interval for 50% randomized of 42.1 to 57.9%.

Our target registration number for this feasibility trial is therefore 200 patients, of which we hope that at least 60 patients will be randomized into the two groups. Thirty patients per arm will provide enough power to create a 95% confidence interval around the risk of VTE recurrence, which would have a width of 34% or less. As an example, if we found that the risk of VTE occurrence was 50% in the arm that stopped, we would be able to estimate a confidence interval of approximately 33% to 67% or smaller. If the risk of VTE were 8%, the confidence interval would be 0 to 19%.

#### RCT data collection

Participants randomized to the RCT will be seen at the recruiting hospital clinical or GP practice (as appropriate) approximately 12 and 26 weeks after starting a further six months treatment with LMWH (treatment arm) or after stopping treatment with LMWH after six months treatment off-trial (control arm). Assessments will include VTE treatment and compliance, physical assessment, full blood count, urea, electrolytes, liver function test, bone profile, toxicities, concomitant medications, use of NHS resources (hospital admissions, GP visits, nurse visits: details to be taken from patient diary booklet and patient notes), and quality of life questionnaires (EORTC QLQ-C30, E5D-5 L and ESAS-r). VTE, bleeding events, and serious adverse events will be collected in real time. Adverse events, in particular the secondary outcomes of recurrent VTE or hemorrhagic events, will be monitored by the Independent Data Monitoring Committee. In the event of excessive bleeding or thrombotic events, this committee will advise on early closure of the trial if necessary. Research staff at recruiting sites will be expected to complete trial case report forms (CRFs) that record evidence of primary and secondary outcome measures. Patients will also be asked for optional consent to have their NHS number collected and to be registered with NHS Information Centre Flagging so that the date and cause of death can be collected without longer term follow up.

#### Statistical analysis

All analyses will be performed on a full intention-to-treat basis, (all patients randomized will be included) and all patients will be analyzed according to their allocated group, whichever treatment they received. We will calculate the percentage of registered patients who were randomized, with 95% confidence interval. We will also calculate the percentage of patients experiencing recurrent VTE recurrence and bleeding events in both groups with 95% confidence intervals. The percentage of patients who died within six months of randomization will be calculated in both arms, along with the percentage of patients who successfully completed six months of trial treatment in the LMWH group. The median QLQ-C30 raw and linear transformed functional scales will be calculated and presented for both arms of the study. No formal subgroup analyses are planned. However, if any treatment effect is found we will investigate whether it is consistent across participant subgroups (defined by all pre-treatment factors collected) although this analysis will be exploratory in nature. Exploratory analyses may be conducted to aid hypothesis generation if a phase three RCT is subsequently developed.

### Embedded qualitative study

To explore clinicians’ attitudes towards the trial and patients’ and their relatives’ experiences of participating in the trial, an embedded qualitative study will be undertaken as part of the larger study. The embedded qualitative study will include focus groups with clinicians and semi-structured interviews with patients and their relatives.

#### Clinician focus groups

Focus groups will be conducted with clinicians from oncology, hematology and primary care settings. Six to ten clinicians will be recruited per group, to enable a variety of perspectives while also ensuring that participants have the opportunity to take part in a dynamic discussion [29]. Two focus groups per clinical setting will be undertaken in order to promote trustworthiness of the findings [29]. Where feasible, for the ease of participants and to not limit the sample to clinicians working in one geographical area [30], focus groups will be held at national meetings and educational events. This has previously been undertaken successfully with clinicians who would otherwise be challenging to recruit to individual interviews of focus groups in other settings [31]. A volunteer sample of clinicians from each clinical setting will be recruited to the focus groups, through advertising the group in the conference literature and via social networking, with clinicians encouraged to contact the researchers or attend on the day.

The focus group will be facilitated by two experienced researchers, with one researcher taking the lead and the other documenting non-verbal communication and ensuring that all participants have the opportunity to participate. It can be challenging to encourage participants to discuss issues relevant to them while ensuring that the research objectives are addressed [29] and therefore a topic guide will be developed that addresses: participants’ attitudes to recruiting to the study in terms of recruitment, equipoise, acceptability of intervention and outcome measures; and participants’ experiences of and attitudes to prescribing LMWH, including whether they would extend treatment past six months. However, the researchers will also encourage participants to explore issues pertinent to them.

#### Semi-structured interviews

This component of the study will explore patients’ and their relatives’ attitudes towards participating in the RCT, potential barriers to and concerns about participation, and factors influencing adherence with self-injection (where appropriate). The most appropriate method for exploring these issues is semi-structured interviews [32], which will be undertaken with: patients who decline randomization, trial participants in the intervention arm, trial participants in the control arm, relatives/carers or trial participants (such as a partner, relative or friend), and participants who withdraw from the intervention or control arm.

In total, 50 to 75 participants will be recruited, including 10 to 15 per group. While this is arguably a large sample size for a qualitative study, it is important to ensure that sufficient numbers of patients are included from each group to promote a variety of perspectives and experiences within the sample. A convenience sample of participants will be recruited from the RCT, a common sampling strategy for embedded qualitative studies within trials [33]. All eligible patients taking part in the RCT will be approached until sufficient numbers have been recruited to represent all groups. Initially, participants will be approached by research practitioners at the recruiting site. When signing the consent form for the RCT component of the study, patients will be able to indicate if they consent to being contacted by the qualitative researcher to discuss being interviewed. The research practitioner will then provide the qualitative researcher with the contact details of consenting participants. The qualitative researcher will contact participants to discuss the interview study, and if the patient agrees, arrange a convenient time and location for interview. LMWH is a home-based treatment and a proportion of relatives assume a caring role in administering the medication. Relatives and/or carers will therefore be recruited by patients taking part in the RCT, enabling patients to have control about who they include [34].

It is anticipated that the interview will take place in the person’s home, for their convenience and for the researcher to be able to speak to the person in their natural environment [35], but can be undertaken in a quiet clinic location if preferred by the participant. The qualitative researcher will take written informed consent immediately prior to the interview where appropriate. Interviews will usually be between 30 and 60 minutes in length, but will be terminated earlier if the participant becomes unwell. Patients who do not consent to participate in the RCT component of the ALICAT study will be interviewed as soon as possible after they have been approached to participate in the trial. ALICAT RCT participants and their relatives/carers will be interviewed after they have completed the treatment phase of the trial, approximately 3 to 12 weeks after the 26 week treatment date. Participants withdrawing from the intervention or control arm of the RCT component of the study will be offered the opportunity to be interviewed at the point of withdrawal.

As with the focus groups, the qualitative interviews will follow a topic guide but also allow participants the flexibility to discuss issues important to them [36]. Patients who take part in the intervention or control arms of the trial will be interviewed to explore: their reasons for, and experiences of, participating in the trial; their views and attitudes towards equipoise; and in the case of the intervention participants, the acceptability of LMWH. Patients who decline randomization will be interviewed to explore their understanding of trial processes, their experiences of the first five months of LMWH off-trial and reasons for non-consent. Participants withdrawing from the study will be interviewed to explore the reasons for their withdrawal and to identify potential strategies and support necessary to minimize attrition. Recruitment to trials in patients with advanced cancer is known to be difficult [37] and patient-reported data in the qualitative arm of this study will inform strategies for recruitment in a full phase three RCT. Relatives/carers of patients who take part in the intervention or control arms of the trial will be interviewed to explore their experiences of caring for someone taking part in the ALICAT trial.

#### Data management

The focus groups and patient interviews will be audio recorded and field-notes will be written to record any instances of non-verbal communication or reaction to any of the discussions. Digital recordings will be transcribed in full and verbatim. Transcripts will be anonymized and subsequently uploaded to QSR NVivo 10 (QSR International) qualitative software for data management and coding.

### Framework analysis

Data will be analyzed to identify problems with systems and procedures that need addressing as well as patient attitudes and experiences. The most appropriate analytic method to achieve this for both the focus group and interview data is framework analysis, an adaptable approach originally developed for use in applied policy research with clear objectives [38]. It is a methodical approach that follows a clear and documented process, but as with other qualitative approaches it relies on the researcher to determine the quality of the analysis [38]. Framework analysis will be carried out in line with Ritchie and Spencer’s five interconnected steps described in Table 4[38].

**Table 4:** **Framework analysis - Ritchie and Spencer’s five interconnected steps**[38]

**Familiarization:**	The researcher will immerse themselves in the data by re-listening to interview recordings and re-reading transcripts and field notes. The researcher will document central ideas and recurring themes.
**Identifying a thematic framework:**	An index of themes will be created, informed by the original research aims around understanding recruitment and retention, but also by issues raised by the participants in the data.
**Indexing the data:**	The index will be applied to each transcript by coding them with the themes from the thematic framework. During this process, the framework may be adjusted, adding new themes and subthemes as they emerge. The adjusted framework will then be applied to subsequent transcripts and reapplied to existing transcripts to ensure all data is appropriately coded.
**Charting:**	A matrix will be created of themes and participants. Data will be lifted from the transcripts and arranged according to thematic references. The data is summarized by the researcher, rather than verbatim quotes included, and referenced back to the original data.
**Mapping and interpretation:**	Charts and research notes will be reviewed to compare and contrast the perceptions and experiences of participants.

The qualitative components of the study (focus groups with clinicians and qualitative interviews with patients and relatives and/or carers) will provide rich data in relation to the attitudes of clinicians recruiting to the study and to patients and their relatives’ motivations to participate in the trial, perceived benefits and burdens, and reasons for withdrawal from the trial. The findings of the qualitative study will be used by the Trial Management Group to assess potential alterations to trial design, and will be used to complement the reporting of the full trial, where appropriate.

### UK wide survey exercise

This component of the study will comprise telephone and web-based surveys to develop a classification and enumeration system for current CAT models and pathways of care. The pilot will be used to identify the relevant variables in the CAT journey and shall be used to develop the most important questions to be asked in the web-based survey. The survey shall then be distributed as widely as possible in order to gain a maximum breadth of understanding pertaining to the patient journey and different service models. Responsibility for the management of patients with ongoing malignancy post-VTE varies across different regions; in some, anticoagulation is managed entirely through the hematology service, whilst some oncology centers will take responsibility for CAT management. Other models are known to exist in which a shared care agreement supports CAT management predominantly in the primary care setting. The survey will be structured around the patient pathway from identification of a VTE, early management and late management developed from the focus groups. We will then undertake a UK wide survey exercise with relevant stakeholders from primary and secondary care. This will initially be in the form of a telephone survey to essentially pilot and clarify the survey tool. The survey will explore the views and experiences of relevant clinical stakeholders involved in the CAT journey from diagnosis to ongoing management, and will include clinicians and allied health professionals from radiology, hematology, oncology primary care and pharmacy. Initial questions shall be designed around the findings from the focus group data in order to conduct semi-structured interviews. These findings shall then inform the design of a more extensive web survey sent out to doctors, nurses and allied health professionals potentially involved in the care of patients with CAT, and will allow for classification and enumeration of the models of care. This will also be triangulated with available documentary evidence on pathways of care. A particular focus will be on the management of transition points between settings and how continuities in care are assured. Respondents will be encouraged to submit any local documents they use on care pathways.

### Outcome measures

The primary outcome measures are: the number of eligible patients identified over 12 months, the number of recruited patients (randomized participants) over 12 months (target recruitment rate of 30% of eligible patients), and the proportion of participants with recurrent VTEs during trial follow up. VTE will be objectively confirmed through radiological investigation. DVT will be confirmed through Doppler ultrasonography or venography. PE shall be confirmed through a computerized tomography (CT) pulmonary angiogram. The secondary outcome measures are: completion of trial protocol, quality of life, symptom assessment, patients’ and relatives’ experiences of taking part in the trial, and the attitudes of clinicians towards the ALICAT trial.

Completion of trial protocol will be assessed six months after randomization to ascertain the attrition rate due to patient choice or death during the study period. Participants choosing to withdraw from either arm of the study protocol will be invited to participate in a qualitative interview to explore their reasons for withdrawal.

Quality of life will be measured using the EORTC QLQ-C30 Version 3.0 and EQ-5D-5 L at three monthly intervals for six months. The EORTC QLQ-C30 [39], Version 3.0, has become a benchmark measure of quality of life in patients with cancer. It contains five functional scales (physical, role, cognitive, emotional and social), three symptom scales (pain, nausea and/or vomiting, and fatigue), global health and quality of life scales, and several other single items. The EQ-5D-5 L [40] is a short quality of life tool, designed to complement other measures and is recommended by the National Institute for Health and Care Excellence for use in economic analyses [41]. The ALICAT study will identify key cost drivers to inform the design of a future definitive phase three trial, which will include a cost utility study. Orders of magnitude of differences in costs and outcomes identified in the EQ-5D-5 L will help in the estimation of anticipated effect sizes for the full trial.

Symptom assessment will be measured using the Edmonton Symptom Assessment System Revised (ESAS-r) at three monthly intervals for six months. The ESAS-r is used to capture patients’ perspective on their symptoms, providing an indication of severity of nine symptoms: pain, tiredness, drowsiness, nausea, lack of appetite, depression, anxiety, shortness of breath, and wellbeing [42,43]. In addition, we will look for symptoms which are likely to be specifically due to VTE (new or worse leg swelling and/or pain, new or worse breathlessness, pleuritic chest pain).

### Ethical, regulatory and management considerations

ALICAT has received ethical and governance approvals from the South East Wales Research Ethics Committee (ref: 13/WA/0026) and NHS sites. The study has been approved to be conducted in the UK by the MHRA. The study sponsor is Cardiff University. The study protocol was written with reference to the Research Government Frameworks for England [44] and Wales [45] and follows the principles of Good Clinical Practice. Researchers collecting data will have the appropriate research passports, permissions and letters of access, where required. Written informed consent will be taken from each participant. All data will be managed and stored in concordance of Data Protection Act 1998 [46].

## Discussion

The management of CAT is well established, yet uncertainty remains with respect to the length of anticoagulation in some populations. There is currently no trial data to inform whether continued anticoagulation is warranted in patients with locally advanced or metastatic cancer after receiving anticoagulation for six months. Despite the lack of trial evidence, many clinicians will continue LMWH and, as such, practice has developed without the evidence to support it. Whilst there is ultimately a need to determine whether such patients should receive indefinite anticoagulation or not, the ALICAT study will first establish whether recruitment to such a trial would be feasible. In order to do this, a complex mixed-methods study consisting of a RCT, embedded qualitative study and UK patient pathways mapping exercise, has been developed with the intention to proceed to a full RCT if the data shows that this is feasible.

### Trial status

The trial opened to recruitment in December 2013.

## Abbreviations

ALICAT: Anticoagulation Length in Cancer Associated Thrombosis; CAT: Cancer Associated Thrombosis; CRF: Case Report Form; DVT: Deep vein thrombosis; ESAS-R: Edmonton Symptom Assessment System Revised; HTA: Health Technology Assessment; IMP: Investigative Medicinal Product; LMWH: Low molecular weight heparin; MHRA: Medicines and Healthcare products Regulatory Agency; NIHR: National Institute for Health Research; NHS: National Health Service; PE: Pulmonary embolus; RCT: Randomized controlled trial; SPC: Summary of Product Characteristics; UK: United Kingdom; VKA: vitamin K antagonist; VTE: Venous thromboembolism.

## Competing interests

TB: has received honoraria from Boehringer Ingelheim, Bayer, Pfizer and Daiichi Sankyo for attending advisory boards. GG: Received clinical trial educational grants (and free drug) and given statistical advice to companies who could potentially be manufacturing the Low Molecular Weight Heparin a site decides to use in the trial. Those organizations are not funding this manuscript. DF: Received honoraria from Bayer, BI, and Sanofi-Aventis. KH: The South East Wales Trials Unit is in receipt of funding from Pfizer for the hosting and maintenance of the TRAD Alliance website & the TROPHY database. ACo: Advisory boards for Bayer, Bristol-Myers Squibb, Daiichi Sankyo, Johnson & Johnson, Pfizer, Portola, and Sanofi. Consulting fees, lecture fees, payment for manuscript preparation, and payment for the development of educational presentations from Astellas, AstraZeneca, Bayer, Boehringer Ingelheim, Bristol-Myers Squibb, Daiichi Sankyo, GlaxoSmithKline, Johnson & Johnson, Mitsubishi Pharma, Pfizer, Portola, Sanofi, Schering Plough, and Takeda. MJ: Co-director of the TRAD Alliance, which is supported by an unrestricted educational grant from Pfizer. AM: Consultancy and advisory boards for Leo and Pfizer. SN: Research grant support from Leo and Pfizer. Consultancy for Leo, Pfizer, Bayer. Speaking honoraria Leo Pharma. The authors declare no other competing interests.

## Authors’ contributions

JS: conception and design, trial management and manuscript writing. JB: study design, will undertake qualitative data collection and analysis, and manuscript writing. TB: conception and design, and contribution to manuscript. GOG: conception and design, and contribution to manuscript. ACa: conception and design, will undertake statistical analysis, manuscript writing and final approval of the manuscript. DC: conception and design, and contribution to manuscript. DF: conception and design, primary care coordination for the trial and contribution to manuscript. KH: conception and design, coordination of the UK survey exercise and manuscript writing. PR: conception and design, coordination of the hematology setting and contribution to manuscript. ACo: conception and design, and contribution to manuscript. MJ: conception and design, manuscript writing. AM: conception and design, and contribution to manuscript. JBe: conception and design, contribution to manuscript. HT: conception and design, contribution to manuscript. AN: conception and design, coordinating the qualitative embedded study and manuscript writing. SIN: study conception and design, Chief Investigator, coordination of the oncology setting and manuscript writing. All authors read and approved the final manuscript.

## Authors’ information

JDS and JBa were lead authors.
